# Data for a meta-analysis of the adaptive layer in adaptive large neighborhood search

**DOI:** 10.1016/j.dib.2020.106568

**Published:** 2020-11-24

**Authors:** Renata Turkeš, Kenneth Sörensen, Lars Magnus Hvattum, Eva Barrena, Hayet Chentli, Leandro C. Coelho, Iman Dayarian, Axel Grimault, Anders N. Gullhav, Çağatay Iris, Merve Keskin, Alexander Kiefer, Richard Martin Lusby, Geraldo Regis Mauri, Marcela Monroy-Licht, Sophie N. Parragh, Juan-Pablo Riquelme-Rodríguez, Alberto Santini, Vínicius Gandra Martins Santos, Charles Thomas

**Affiliations:** a Department of Mathematics and Computer Science, University of Antwerp, Belgium; b Department of Engineering Management, University of Antwerp, Belgium; c Faculty of Logistics, Molde University College, Norway; d Department of Economics, Pablo de Olavide University, Spain; e Department of Operations Research, University of Science and Technology Houari Boumediene, Algeria; f Operations and Decision Systems Department, Université Laval, Canada; g Department of Information Systems, Statistics, and Management Science, University of Alabama, Alabama; h LARIS, Université d’Angers, France; i Department of Industrial Economics and Technology Management, Norwegian University of Science and Technology, Norway; j Management School, University of Liverpool, England; k Warwick Business School, University of Warwick, England; l Department of Business Decisions and Analytics, University of Vienna, Austria; m Department of Technology, Management, and Economics, DTU, Denmark; n Department of Computing, Federal University of Espírito Santo, Brazil; o DeGroote School of Business, McMaster University, Canada; p Institute of Production and Logistics Management, Johannes Kepler University, Austria; q Escuela de Ingeniería, Universidad Anáhuac, Mexico; r Department of Economics and Business, Universitat Pompeu Fabra, Spain; s Universidade Federal de Ouro Preto, Brazil; t Institute of Information and Communication Technologies, Electronics and Applied Mathematics, UCLouvain

**Keywords:** Meta-analysis, Metaheuristics, Adaptive large neighborhood search

## Abstract

Meta-analysis, a systematic statistical examination that combines the results of several independent studies, has the potential of obtaining problem- and implementation-independent knowledge and understanding of metaheuristic algorithms, but has not yet been applied in the domain of operations research. To illustrate the procedure, we carried out a meta-analysis of the adaptive layer in adaptive large neighborhood search (ALNS). Although ALNS has been widely used to solve a broad range of problems, it has not yet been established whether or not adaptiveness actually contributes to the performance of an ALNS algorithm. A total of 134 studies were identified through Google Scholar or personal e-mail correspondence with researchers in the domain, 63 of which fit a set of predefined eligibility criteria. The results for 25 different implementations of ALNS solving a variety of problems were collected and analyzed using a random effects model. This dataset contains a detailed comparison of ALNS with the non-adaptive variant per study and per instance, together with the meta-analysis summary results. The data enable to replicate the analysis, to evaluate the algorithms using other metrics, to revisit the importance of ALNS adaptive layer if results from more studies become available, or to simply consult the ready-to-use formulas in the summary file to carry out a meta-analysis of any research question. The individual studies, the meta-analysis and its results are described and interpreted in detail in Renata Turkeš, Kenneth Sörensen, Lars Magnus Hvattum, Meta-analysis of Metaheuristics: Quantifying the Effect of Adaptiveness in Adaptive Large Neighborhood Search, in the European Journal of Operational Research.

## Specifications Table

**Table:** 

Subject	Management Science and Operations Research
Specific subject area	Analysis of metaheuristic algorithms
Type of data	Table
How data were acquired	For each individual study included in the meta-analysis, the ALNS and its non-adaptive variant (¬A)LNS were run a number of times on a number of problem instances. These results from the individual studies were pre-processed and then analyzed with a random-effects model.
Data format	Raw Filtered Analyzed
Parameters for data collection	We performed a literature review of ALNS, restricting our search to articles that: • describe the weight adjustment mechanism used in sufficient detail, • employ a weight adjustment formula which includes a parameter, that could be set to a certain value so that the adaptive layer is switched off, and • employ a roulette wheel mechanism to choose between heuristics.
Description of data collection	The results of the comparison of ALNS and (¬A)LNS for [1], [2] are directly obtained from the respective articles. The results from the remaining individual studies were collected via e-mail. For every individual study $S_{k},$ we calculate the added value $A_{k}$ of the ALNS adaptive layer, and the within-study variance $V_{k},$ which are then analyzed with a random effects model to obtain the summary importance A of adaptiveness across all included studies.
Data source location	Institution: University of Antwerp City/Town/Region: Antwerp Country: Belgium
Data accessibility	Repository name: Mendeley Data, Turkeš, Renata (2020), ”Data for a meta-analysis of the adaptive layer in Adaptive Large Neighborhood Search” Data identification number: 10.17632/h4smx32r4t.3 Direct URL to data: https://doi.org/10.17632/h4smx32r4t.3
Related research article	Renata Turkeš, Kenneth Sörensen, Lars Magnus Hvattum, Meta-analysis of Metaheuristics: Quantifying the Effect of Adaptiveness in Adaptive Large Neighborhood Search, European Journal of Operational Research, https://doi.org/10.1016/j.ejor.2020.10.045[3]

## Value of the Data

• Detailed per-instance comparison results of ALNS with its non-adaptive variant across a number of independent studies, i.e., implementations of ALNS to solve a broad range of different problems, helps to evaluate the importance of ALNS adaptive layer.
• The data are of greatest interest for researchers interested in Adaptive Large Neighborhood Search, and in particular its adaptive layer. Furthermore, the ready-to-use sheet with random effects model formulas can benefit those interested in carrying out a meta-analysis of any research question within operations research, or any domain.
• The data enable to replicate the analysis, to evaluate the algorithms using other metrics, to study the influence of different factors on the added value of ALNS adaptive layer, to revisit its importance if results from more studies become available, or to simply consult the summary file for a meta-analysis of any research question.

## 1 Data Description

In adaptive large neighborhood search (ALNS), a solution is iteratively destroyed and repaired through the application of several heuristics h∈H. In order to select the heuristic to use, a weight is assigned to each destroy heuristic h∈D and each repair heuristic h∈R. First, weights are set to some initial values, which are usually equal. An adaptive weight adjustment procedure updates these weights based on the performance of each heuristic. At the end of each segment s (a number of iterations), the weight $w_{h}^{s+1}$ of the heuristic h is calculated as follows:

(1) $$w_{h}^{s+1}=\left(1-r\right)w_{h}^{s}+r\frac{\pi_{h}}{\theta_{h}},$$

where $\pi_{h}$ is the score of heuristic which reflects its performance, $\theta_{h}$ is the number of times heuristic h was used during the last segment, and r is the reaction factor. The reaction factor r controls how quickly the weight adjustment procedure reacts to changes in the effectiveness of the heuristic, and if r=0, the weights remain unchanged.

This dataset contains a detailed comparison of ALNS and its non-adaptive variant (¬A)LNS, for ALNS implementations described in [1], [2], [4], [5], [6], [7], [8], [9], [10], [11], [12], [13], [14], [15], [16], [17], [18], [19], [20], [21], [22]. In other words, it lists the results of comparing ALNS with the value of the reaction factor r as chosen in each of the articles, and ALNS with r=0 (without adaptiveness). The results from the individual studies are then processed, and analyzed with a random effects model. The dataset is structured into two folders and a summary file:

• data_individiual_studies_raw.zip
• data_individiual_studies_filtered.zip
• data_analyzed.xls

The folder data_individiual_studies_raw.zip consists of the comparison of ALNS with the non-adaptive variant per study and per instance, in the format (.xslx,.xsl,.csv,.ods,.xml or.html) summarized by the authors of the individual studies and e-mailed to Renata Turkeš, and is only added for the purpose of completeness and transparency.

The folder data_individiual_studies_filtered.zip consists of the comparison of ALNS with the non-adaptive variant per study and per instance, and corresponds to the raw data from data_individiual_studies_raw.zip, but pre-processed by Renata Turkeš. Each of the files corresponding to an individual study $S_{k}$ is specified in the same format, the redundant data are removed, and some further information is calculated in order to summarize the impact of the ALNS adaptive layer for each study.

More precisely, a file corresponding to study $S_{k}$ starts with the information about the article title and objective function $f_{k},$ with the main information summarized in a table. The first three table columns list instance names, and the average objective function value across a number of runs of the best solution found by ALNS and its non-adaptive variant, and are obtained from data_individiual_studies_raw.zip. These objective function values are then used to calculate the next four columns, which evaluate the improvement in the objective function value with the adaptive layer, and whether ALNS outperforms (¬A)LNS or not. Finally, from this table we calculate some summary values for the considered study: average, variance and 95% confidence interval for the added value of the ALNS adaptive layer, across problem instances. The calculation of the improvement $A_{k}$ in the objective function value with adaptiveness in study $S_{k},$ and the within-study variance $V_{k}$ is described in great detail in the next section on the experimental design. For example, the file grimault2017adaptive.xlsx corresponding to the ALNS introduced in [7] is summarized in Table 1.

**Table 1:** Example of a file in data_individiual_studies_filtered.zip, summarizing the importance of the adaptive layer for a single individual study $S_{k}$.

Article title: An adaptive large neighborhood search for the full truckload pickup and delivery problem with resource synchronization						
Minimization problem (minimization of the sum of travel costs, costs of service times and vehicle utilization costs)						
instance I	objective function value (¬A)LNS with reaction factor (r=0), averaged across 10 runs $\bar{f_{k}}\left(x_{0}\left(I\right)\right)$	objective function value ALNS with reaction factor $(r=r_{k}=0.5),$ averaged across 10 runs $\bar{f_{k}}\left(x_{r_{k}}\left(I\right)\right)$	improvement A in objective function value with adaptiveness, averaged across 10 runs, $\frac{\bar{f_{k}}\left(x_{r_{k}}\left(I\right)\right)-\bar{f_{k}}\left(x_{0}\left(I\right)\right)}{\bar{f_{k}}\left(x_{0}\left(I\right)\right)}$(%) (minimization problem)	ALNS better than (¬A)LNS	ALNS worse than (¬A)LNS	ALNS equal to (¬A)LNS
OS22	2786.93	2797.92	-0.39	0	1	0
OS30	4840.30	4848.06	-0.16	0	1	0
OS49	6433.89	6414.43	0.30	1	0	0
		improvement $A_{k}$ in objective function value with adaptiveness (%), averaged across runs and instances	-0.08	1	2	0
		standard deviation $\sigma_{k}$	0.35	% better:	% worse:	% equal:
		number of instances $N_{k}$	3	33.33	66.67	0
		within-study variance $V_{k}=\frac{\sigma^{2}}{N_{k}}$	0.04			
		standard error $\frac{\sigma_{k}}{\sqrt{N_{k}}}$	0.20			
		95% confidence interval lower bound	-0.48			
		95% confidence interval upper bound	0.32			

The folder data_individiual_studies_filtered.zip thus consists of 21 files in.xlsx format for each of the 21 individual studies included in the meta-analysis. A few of the files for some of the individual studies consist of a number of separate sheets, corresponding to the different ALNS versions considered, or for multiple instance classes.

Finally, these results from the individual studies are used for the meta-analysis of the ALNS adaptive layer, available in the table in data_analyzed.xls. The summary effect A reflecting the importance of the adaptive layer is the weighted average of effects $A_{k}$ of individual studies. The study weights $W_{k}$ are calculated using the within-study variance $V_{k}$ and the variance $T^{2}$ across studies. Table 2 lists the features that are calculated for each study, as described in detail in the next section on the experimental design.

**Table 2:** The columns in data_analyzed.xls correspond to features obtained for each study, which are then used to calculate the importance of the adaptive layer across all studies.

article, i.e., study $S_{k}$	Data obtained from data_individiual_studies_filtered.zip.
observed effect $A_{k}$	
within-study variance $V_{k}=\frac{\sigma_{k}^{2}}{N_{k}}$	
$\frac{1}{V_{k}}$	Auxiliary columns to calculate variance $T^{2}$ across studies.
$\frac{1}{V_{k}}A_{k}$	
$\frac{1}{V_{k}}A_{k}^{2}$	
${\left(\frac{1}{V_{k}}\right)}^{2}$	
between study variance $T^{2}$	
weight $W_{k}=\frac{1}{V_{k}+T^{2}}$	
normalized weight $\frac{W_{k}}{\sum_{j}W_{j}}$	
weighted effect $\frac{W_{k}}{\sum_{j}W_{j}}A_{k}$	

## 2 Experimental Design, Materials and Methods

The summary effect A reflecting the importance of the adaptive layer is the weighted average of effects $A_{k}$ of individual studies $S_{k},$ k∈{1,2,3,⋯,K}.

Let us assume study $S_{k}$ considers a maximization problem with the objective function $f_{k},$ and let $I_{k}=\left\{I_{1}^{k},I_{2}^{k},\cdots ,I_{N_{k}}^{k}\right\}$ denote the set of available problem instances. We run ALNS introduced in study $S_{k},$ with the value of the reaction factor $r_{k}$ chosen in the individual article, to find the solution $x_{r_{k}}^{*}\left(I\right)$ for problem instance $I\in I_{k}.$ The best solution found by the non-adaptive (¬A)LNS with r=0 for the same problem instance is denoted with $x_{0}^{*}\left(I\right).$ Since ALNS is not a deterministic algorithm, we run both algorithms several times on each problem instance, and calculate the average objective function values across a number of runs, $\bar{f_{k}}\left(x_{r_{k}}^{*}\left(I\right)\right)$ and $\bar{f_{k}}\left(x_{0}^{*}\left(I\right)\right).$ The added value of adaptiveness in study $S_{k}$ is calculated as the improvement in the average objective function value yielded with the adaptive layer, across the set of available instances:

(2) $$A_{k}=\frac{1}{N_{k}}\sum_{I\in I_{k}}\frac{\bar{f_{k}}\left(x_{r_{k}}^{*}\left(I\right)\right)-\bar{f_{k}}\left(x_{0}^{*}\left(I\right)\right)}{\bar{f_{k}}\left(x_{0}^{*}\left(I\right)\right)}.$$

If we are considering a minimization problem, the average improvement in the objective function for study $S_{k}$ is calculated as

(3) $$A_{k}=-\frac{1}{N_{k}}\sum_{I\in I_{k}}\frac{\bar{f_{k}}\left(x_{r_{k}}^{*}\left(I\right)\right)-\bar{f_{k}}\left(x_{0}^{*}\left(I\right)\right)}{\bar{f_{k}}\left(x_{0}^{*}\left(I\right)\right)}.$$

The weight $W_{k}$ of study $S_{k}$ is calculated as inverse variance. In a random effects model, variance is calculated as the sum of within-study variance and variance across studies. The within-study variance is estimated with the squared standard error:

$$V_{k}=\frac{\sigma_{k}^{2}}{N_{k}},$$

($\sigma_{k}$ is the standard deviation, and $N_{k}$ is the number of problem instances in study $S_{k}$). It makes sense to weigh studies with the inverse variance: we assign more weight to the studies which include a greater number of instances, and for which the dispersion of the effect size across instances is small.

The between-study variance is estimated using the DerSimonian and Laird method:

$$T^{2}=\frac{Q-df}{C},$$

where:

• Q is a sum of squares of the effect size estimates about their mean, weighted by the inverse of variance $V_{k},$
  $$Q=\sum_{k}\frac{1}{V_{k}}{\left(A_{k}-\frac{1}{\sum_{j}\frac{1}{V_{j}}}\sum_{j}\frac{1}{V_{j}}A_{j}\right)}^{2}=\sum_{k}\frac{1}{V_{k}}A_{k}^{2}-\frac{1}{\sum_{k}\frac{1}{V_{k}}}{\left(\sum_{k}\frac{1}{V_{k}}A_{k}\right)}^{2},$$
• df degrees of freedom, df=K−1, where K is the number of studies included in the meta-analysis,
• C is simply a factor which puts the standardized variation between studies Q−df back into the same metric that had been used to report the within-study variance,
  $$C=\sum_{k}\frac{1}{V_{k}}-\frac{1}{\sum_{k}\frac{1}{V_{k}}}\sum_{k}\frac{1}{V_{k}^{2}}.$$

If $T^{2}$ is less than zero, it is set to zero, since variance cannot be negative.

The total variance under the random effects model is therefore $V_{k}+T^{2},$ so that the weight of study $S_{k}$ is calculated as:

$$W_{k}=\frac{1}{V_{k}+T^{2}}.$$

In the remainder of this section, we illustrate the experimental design (i.e., all the calculations carried out in data_individiual_studies_filtered.zip and data_analyzed.xls) with a small example of a meta-analysis with only two studies, with two and three considered problem instances, and two runs of the algorithms for each instance, summarized in Table 3. The information in the highlighted upper-left rectangle corresponds to data collected from a single study $S_{k}:$ the objective function value of the best solution found by ALNS and the non-adaptive variant (¬A)LNS, for a number of problem instances and algorithmic runs. This information is used to estimate the mean importance $A_{k}$ of the adaptive layer, i.e., the average improvement of ALNS upon the non-adaptive algorithm, for a study $S_{k}$.

**Table 3:** A toy example of a step-by-step meta-analysis with two included studies.

Study $S_{k}$	Instance $I\in I_{k}$	Run	$f_{k}\left(x_{0}^{*}\left(I\right)\right)$	$f_{k}\left(x_{r_{k}}^{*}\left(I\right)\right)$	$\bar{f_{k}}\left(x_{0}^{*}\left(I\right)\right)$	$\bar{f_{k}}\left(x_{r_{k}}^{*}\left(I\right)\right)$	$\frac{\bar{f_{k}}\left(x_{r_{k}}^{*}\left(I\right)\right)-\bar{f_{k}}\left(x_{0}^{*}\left(I\right)\right)}{\bar{f_{k}}\left(x_{0}^{*}\left(I\right)\right)}$(%)	Effect $A_{k}$(%)	Standard deviation $\sigma_{k}$	Number of instances $N_{k}$	Within-study variance $V_{k}=\frac{\sigma_{k}^{2}}{N_{k}}$	Between-study variance $T^{2}$	Weight $W_{k}=\frac{1}{V_{k}+T^{2}}$
$S_{1}$	$I_{1}^{1}$	1	856.0	863.0	855.00	866.50	1.35		0.50	2	0.13	0	
		2	854.0	870.0									
	$I_{2}^{1}$	1	40.0	39.0	39.00	39.25	0.64						
		2	38.0	39.5				1.00					7.93
$S_{2}$	$I_{1}^{2}$	1	1200.0	1208.0	1200.00	1206.5	0.64		3.26	3	3.55		
		2	1200.0	1205.0									
	$I_{2}^{2}$	1	10.0	10.5	10.5	11.1	5.71						
		2	11.0	11.7									
	$I_{3}^{2}$	1	301.0	299.0	300.5	299.5	-0.33						
		2	300.0	300.0				1.97					0.28

The weights $W_{k}$ of the studies are then calculated as the sum of within-study variance $V_{k}$ (square of standard error, which incorporates both the standard deviation $\sigma_{k}$ across problem instances within a study, and a number of instances $N_{k}$) and between-study variance $T^{2}$ (estimated with poor precision when the number studies is very small). The summary effect A in this example is weighted more strongly towards $A_{1}$ than $A_{2},$ since the weight of the study $S_{2}$ is very small: indeed, the adaptive layer improves the algorithmic performance by 0.64% for one instance, 5.71% for another instances, but by −0.33% for the last instance, and we are therefore less confident about the true effect of adaptiveness in this study (i.e., the standard deviation is large, and hence the weight is small, indicating that the estimate $A_{2}$ is less precise).

The summary effect A reflecting the importance of the adaptive layer for the two small studies $S_{1}$ and $S_{2}$ is the weighted average of effects $A_{1}$ and $A_{2}:$

$$A=\frac{W_{1}}{W_{1}+W_{2}}\times A_{1}+\frac{W_{2}}{W_{1}+W_{2}}\times A_{2}=\frac{7.93}{7.93+0.28}\times 1.00\%+\frac{0.28}{7.93+0.28}\times 1.97\%=1.03\%.$$

## Author contributions

Renata Turkeš carried out the literature review, identified and selected the studies, communicated with the authors of eligible studies via e-mail, collected and pre-processed the raw data into data_individiual_studies_filtered.zip, and wrote the manuscript. These results from individual studies were then analyzed by Renata Turkeš, Kenneth Sörensen and Lars Magnus Hvattum, and summarized in data_analyzed.zip. Eva Barrena, Hayet Chentli, Leandro Coelho, Iman Dayarian, Axel Grimault, Anders Gullhav, Çağatay Iris, Merve Keskin, Alexander Kiefer, Richard Lusby, Geraldo Mauri, Marcela Monroy-Licht, Sophie N. Parragh, Juan-Pablo Riquelme-Rodríguez, Alberto Santini, Vinicius Gandra Martins Santos and Charles Thomas carried out the experiments which compare an ALNS previously introduced in an individual study, with its non-adaptive variant, collected in data_individiual_studies_raw.zip.

## Declaration of Competing Interest

The authors declare that they have no known competing financial interests or personal relationships which have, or could be perceived to have, influenced the work reported in this article.

## Figures and Tables

**Table A1:** Important notation and acronyms.

Acronyms	
ALNS	adaptive large neighborhood search
(¬A)LNS	ALNS without the adaptive layer
**Notation**	
H	set of (destroy or repair) heuristics
D	set of destroy heuristics
R	set of repair heuristics
h	(destroy or repair) heuristic
s	segment, a number of consecutive iterations
$\pi_{h}$	total score of heuristic h at the end of the current segment
$\theta_{h}^{s}$	number of times heuristic h has been called during the current segment
r	reaction factor, which controls the importance of heuristic performance in the last segment
$w_{h}^{s}$	weight of heuristic h updated after segment s (calculated as $\left(1-r\right)w_{h}^{s-1}+r\pi_{h}/\theta_{h}$)
K	number of studies in the meta-analysis
$S_{k}$	study $S_{k}$ (an individual study in the meta-analysis)
$f_{k}$	objective function in study $S_{k}$
$\bar{f_{k}}\left(\cdot \right)$	average objective function value in study $S_{k}$ across a number of runs
$x_{r_{k}}^{*}$	best solution found by ALNS from study $S_{k},$ with reaction factor $r_{k}$
$x_{0}^{*}$	best solution found by non-adaptive (¬A)LNS, with r=0
$I_{i}^{k}$	instance $I_{i}$ in study $S_{k}$
$A_{k}$	improvement with the adaptive layer in study $S_{k},$ averaged across instances and runs
$N_{k}$	number of instances in study $S_{k}$
$\sigma_{k}$	standard deviation in study $S_{k}$
$V_{k}$	variance within study $S_{k}$ (calculated as $\sigma_{k}^{2}/N_{k})$
$T^{2}$	variance across studies
$W_{k}$	weight of study $S_{k}$ (calculated as inverse total variance $1/(V_{k}+T^{2})$)
A	improvement with the adaptive layer, weighted across studies

## Appendix A

Table A.4 summarizes the notation.
